# Elevated circulating PCSK9 level is associated with 28-day mortality in patients with sepsis: a prospective cohort study

**DOI:** 10.1186/s12873-023-00896-6

**Published:** 2023-10-31

**Authors:** Yuanlu Shu, Ziwei Deng, Ye Deng, Jianliang Zhou, Jin Wang, Zhenxing Duan, Tao Jiang, Xiang Zhao, Zhihua Shi, Chengfeng Qiu

**Affiliations:** 1 Evidence-based Medicine and Clinical Center, The First People’s Hospital of Huaihua, Huaihua, 418000 P.R. China; 2 Clinical Pharmacy Department, The First People’s Hospital of Huaihua, Huaihua, 418000 P.R. China; 3 Emergency Department, The First People’s Hospital of Huaihua, Huaihua, 418000 P.R. China; 4 School of Basic Medical Sciences, University of South China, Hengyang, 421000 P.R. China

**Keywords:** PCSK9, Mortality, Sepsis

## Abstract

**Objectives:**

Pro-protein convertase subtilisin/kexin 9 (PCSK9) decreases the clearance of the pathogenic lipids, supporting the potential role of PCSK9 in the prognosis of sepsis.

**Methods:**

In this prospective cohort study, patients with sepsis were consecutively recruited from 1 to 2020 to 30 September 2021 at the First People’s Hospital of Huaihua, China. All the eligible patients were categorized into low-PCSK9 and high-PCSK9 groups, based on their PCSK9 levels at admission. Time-dependent receiver operating characteristic curves and Cox proportional hazards regression were used to evaluate the association between PCSK9 level and 28-day mortality of sepsis.

**Results:**

Of the 203 enrolled patients, 56 (27.59%) died during the 28-day follow-up. The PCSK9 level was positively related to the C-reactive protein level. The cut-off point of PCSK9 levels for 28-day mortality risk was 370 ng/ml. Through comparison between high-PCSK9 (> 370 ng/ml) with low-PCSK9 (≤ 370 ng/ml) groups, the adjusted HR for mortality was 2.56 (95% CI: 1.25–5.23, *p* = 0.01).

**Conclusions:**

The 28-day mortality of sepsis increased significantly as the baseline circulating PCSK9 level exceeded 370 ng/ml, indicating circulating PCSK9 levels may be a potential biomarker to predict the prognosis of sepsis.

**Supplementary Information:**

The online version contains supplementary material available at 10.1186/s12873-023-00896-6.

## Introduction

Sepsis, a life-threatening organ dysfunction syndrome, arises as the host response to infection is dysregulated. Despite emerging treatments for sepsis, mortality remains high in the intensive care unit [1, 2]. Extensive studies have confirmed that cholesterol and pathogenic lipids are closely involved in the pathogenesis of sepsis.

Cholesterol and pathogenic lipids trigger the inflammatory process via signaling Toll-like receptors, which then augment the production of cytokines [3]. Increasing the clearance of circulating cholesterol and pathogenic lipids may improve patient’s clinical outcomes [4]. Hepatic low-density lipoprotein receptor (LDLR) plays a key role in determining the level of plasma cholesterol by mediating its clearance from circulation [5]. LDLR also represses the inflammatory response through clearing pathogenic lipids [5, 6].

Proprotein convertase subtilisin/kexin type-9 (PCSK9), a circulating protein, binds to LDLR on hepatocytes’ surface and targets it for lysosomal degradation [7]. Due to this capability, PCSK9 has become a promising cholesterol-lowering candidate [8]. Furthermore, a low density of hepatic LDLR may hinder the clearance of cholesterol and pathogenic lipids from circulation [9]. Evidence has also demonstrated that PCSK9 is closely related to inflammation, with a serum level increasing significantly during sepsis [9, 10].

Nowadays, genetic and clinical trials have explored whether circulating PCSK9 is a prognosis biomarker of sepsis [10–14]. Unfortunately, their results are inconsistent, and can’t reach a clear-out conclusion. Here conducted a study on Chinese patients to identify the relationship between the circulating PCSK9 level and the 28-day mortality of sepsis.

## Materials and methods

### Study design and participants

This study was a prospective single-center, observational cohort study conducted at the First People’s Hospital of Huaihua, a treatment center for critically ill patients in Huaihua, China. The study was conducted in January 2020. Participants were recruited from 1 to 2020 to 30 September 2021. Patients aged 18 years or older and meeting the clinical criteria for Sepsis 3.0 within 24 h before any time point during their stay in the hospital were enrolled [15]. The exclusion criteria were as follows: (i) more than 24 h had passed since the patient was defined as meeting inclusion criteria; (ii) the patient was in a terminal state or died within 48 h; (iii) the patient showed inability or refusal to sign informed consent.

Follow-up time was defined as the period from the establishment of diagnosis to the onset of death, loss to follow-up, or the end of follow-up (28 days).

The study was approved by the research ethics committee of the First People’s Hospital of Huaihua (No. KY-2,019,082,203). Informed consent was obtained from all study participants. The study was carried out in accordance with relevant guidelines and regulations (e.g. Declaration of Helsinki). The study protocol was available at http://www.chictr.org.cn (ChiCTR1900026452).

### Data collection and PCSK9 detection

Within 24 h after admission, demographic and clinical characteristics of the patients, including age, sex, and underlying diseases, were collected by researchers. The data collection of laboratory results were defined using the first-time examination at admission (within 6 h after admission).

Venous blood samples were collected in sterile Ethylene Diamine Tetraacetic Acid (EDTA)-coated tubes within 1 h after diagnosis, during which any drug use was avoided. Plasma was collected by centrifugation at 1000 g for 15 min at room temperature, pipetted into Eppendorf tubes, and stored at − 80 °C. PCSK9 was measured using a colorimetric enzyme-linked immunosorbent assay (ELISA) according to the manufacturer’s instructions (Cusabio, Wuhan, China).

### Statistical analysis

Continuous variables were presented as the median and interquartile range (IQR), and categorical variables as numbers and percentages. Continuous variables were compared using the Wilcoxon rank-sum test and categorical using the chi-square test. Cross-sectional associations between PCSK9 levels and other characteristics were analyzed using Pearson correlation coefficients.

Time-dependent receiver operating characteristic (ROC) curve analysis determined the optimum threshold value of PCSK9. Cumulative event survival curves for 28-day mortality were compared across dichotomous categories of PCSK9 using the log-rank test. Cox proportional-hazards regression models were used to calculate covariate-adjusted hazard ratios (HRs) of PCSK9 for predicting the risk of 28-day mortality.

The *p*-value was 2-sided, and an alpha level of 0.05 was used to define statistical significance. All analyses were conducted using R software (version R 3.6.3; https://cran.r-project.org/).

## Result

### Patient characteristics

**Fig. 1:**
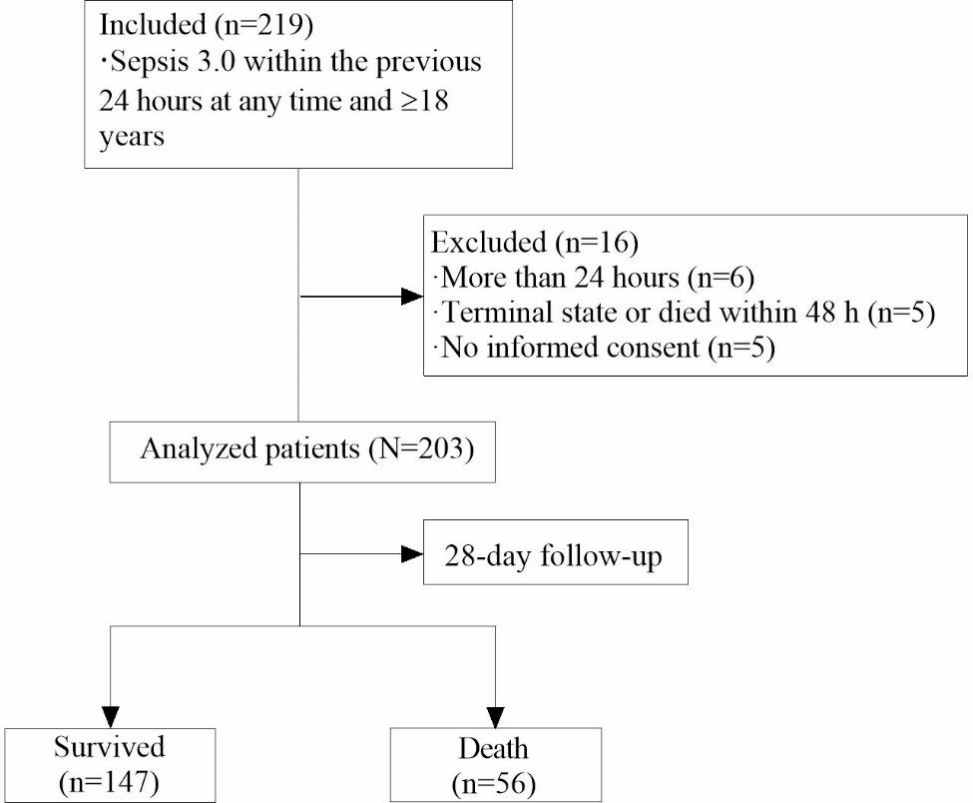
Study flowchart

Figure 1 shows the flowchart of patient recruitment. The demographic and clinical characteristics of the 203 patients are summarized in Table 1. The median age was 63.00 years (IQR, 51.50–72.50), and 123 (60.59%) patients were male. The median PCSK9 level was 381.60 (IQR 223.50-655.80) ng/ml. During the 28-day follow-up, 56 (27.59%) patients died. The PCKS9 levels in those who died than in those who still survived (median [IQR]: 568.30 [345.80, 756.00] ng/ml vs. 343.50 [202.90, 602.20] ng/ml).

**Table 1:** Baseline characteristics of study patients

Variables	Total (n = 203)	Survival(N = 147)	Death(N = 56)	*p*
**Demographic**				
Age, Median (IQR)	63.00 (51.50, 72.50)	62.00 (51.00, 73.00)	63.00 (54.00, 72.00)	0.95
Male sex, n (%)	123 (60.59)	85 (57.82)	38 (67.86)	0.25
**Comorbidities**, n (%)				
Hypertension	61 (30.05)	39 (26.53)	22 (39.29)	0.11
Diabetes	68 (33.50)	46 (31.29)	22 (39.29)	0.36
CHD	48 (23.65)	33 (22.45)	15 (26.79)	0.52
CKD	32 (15.76)	21 (14.28)	11 (19.64)	0.35
Liver disease	28 (13.79)	18 (12.24)	10 (17.86)	0.30
cerebrovascular disease	16 (7.88)	9 (6.12)	7 (12.50)	0.22
Tumour	0 (0)	0 (0)	0 (0)	
**SOFA score ≥ 2**, n (%)				
Cardiovascular	76 (37.44)	32 (21.77)	44 (78.57)	< 0.001
Coagulation	69 (33.99)	49 (33.33)	20 (35.71)	0.75
Liver	41 (20.20)	22 (14.97)	19 (33.93)	0.003
Neurologic	53 (26.11)	24 (16.33)	29 (51.79)	< 0.001
Renal	55 (27.09)	36 (24.49)	19 (33.93)	0.18
Respiratory	78 (38.42)	40 (27.21)	38 (67.86)	< 0.001
**SOFA**, Median (IQR)	5.00 (4.00, 8.00)	4.00 (3.00, 6.00)	9.00 (7.00, 11.00)	< 0.001
**Biochemistry**, Median (IQR)				
ALT, IU/L	54.00 (33.00, 82.00)	45.00 (28.00, 71.00)	59.50 (37.00, 85.50)	0.01
AST, IU/L	41.00 (26.00, 100.00)	40.00 (27.50, 96.00)	42.50 (25.00, 104.00)	0.67
Total bilirubin, µmol/L	14.94 (9.67, 25.54)	15.08 (10.40, 24.02)	12.91 (9.48, 32.34)	0.85
Albumin, g/L	29.50 (26.40, 32.40)	29.90 (25.20, 32.65)	28.90 (26.72, 30.13)	0.09
Creatinine, µmol/L	151.00 (92.00, 232.00)	135.00 (83.00, 184.00)	193.00 (97.25, 362.00)	0.004
UA, µmol/L	376.00 (248.00, 503.00)	385.00 (249.00, 503.50)	320.00 (242.00, 503.00)	0.41
BUN, mmol/L	13.04 (7.21, 16.95)	11.67 (7.13, 16.76)	14.21 (8.99, 21.84)	0.03
RBC count, ×10^9^/L	3.60 (3.00, 4.20)	3.60 (3.05, 4.20)	3.10 (2.80, 4.33)	0.11
Hemoglobin, g/L	111.00 (90.00, 126.00)	112.00 (92.50, 125.00)	96.00 (72.00, 125.00)	0.02
WBC count, ×10^9^/L	11.60 (8.35, 21.00)	11.40 (8.00, 20.95)	12.20 (8.78, 23.00)	0.25
Platelet count, ×10^9^/L	146.00 (51.50, 223.00)	145.00 (49.00, 223.00)	150.00 (76.75, 213.00)	0.74
CRP, mg/L	106.10 (73.49, 126.60)	107.30 (71.10, 126.60)	105.70 (82.02, 126.30)	0.63
PCT, µg/L	24.04 (10.90, 35.50)	24.36 (11.18, 34.90)	20.48 (9.27, 35.68)	0.87
Lactate, mmol/L	3.53 (2.34, 5.16)	2.97 (1.90, 4.69)	5.17 (4.15, 8.28)	< 0.001
TC, mmol/L	2.91 (2.08,3.59)	2.93 (2.12, 3.67)	2.89 (1.97, 3.38)	0.15
LDL-C, mmol/L	1.42 (0.95,1.98)	1.40 (0.93, 2.02)	1.49 (1.06, 1.87)	0.58
HDL-C, mmol/L	0.83 (0.51,1.14)	0.80 (0.47, 1.34)	0.94 (0.62, 1.18)	0.13
TG, mmol/L	1.25 (0.75,1.83)	1.37 (0.77, 1.97)	1.09 (0.63, 1.54)	0.03
**Bacteriology**, n (%)				0.45
Gram-positive	101 (49.75)	74 (50.34)	27 (48.21)	
Gram-negative	84 (41.38)	58 (39.46)	26 (46.43)	
Others	18 (8.87)	15 (10.20)	3 (5.36)	
**PCSK9**, ng/ml	381.60 (223.50,655.80)	343.50 (202.90, 602.20)	568.30 (345.80, 756.00)	< 0.001

ALT: alanine aminotransferase; AST: aspartate aminotransferase; BUN: blood urea nitrogen; CHD: coronary heart disease; CKD: chronic kidney disease; CRP: C-reactive protein; HDL-C: high-density lipoprotein cholesterol; LDL-C: low-density lipoprotein cholesterol; PCSK9: proprotein convertase subtilisin/kexin type-9; PCT: procalcitonin; RBC red blood cell; SOFA: Sequential Organ Failure Assessment; TC: total cholesterol; TG: triglyceride; UA: uric acid; WBC: white blood cells

### Correlation between circulating PCSK9 and infection with lipid indexes

**Fig. 2:**
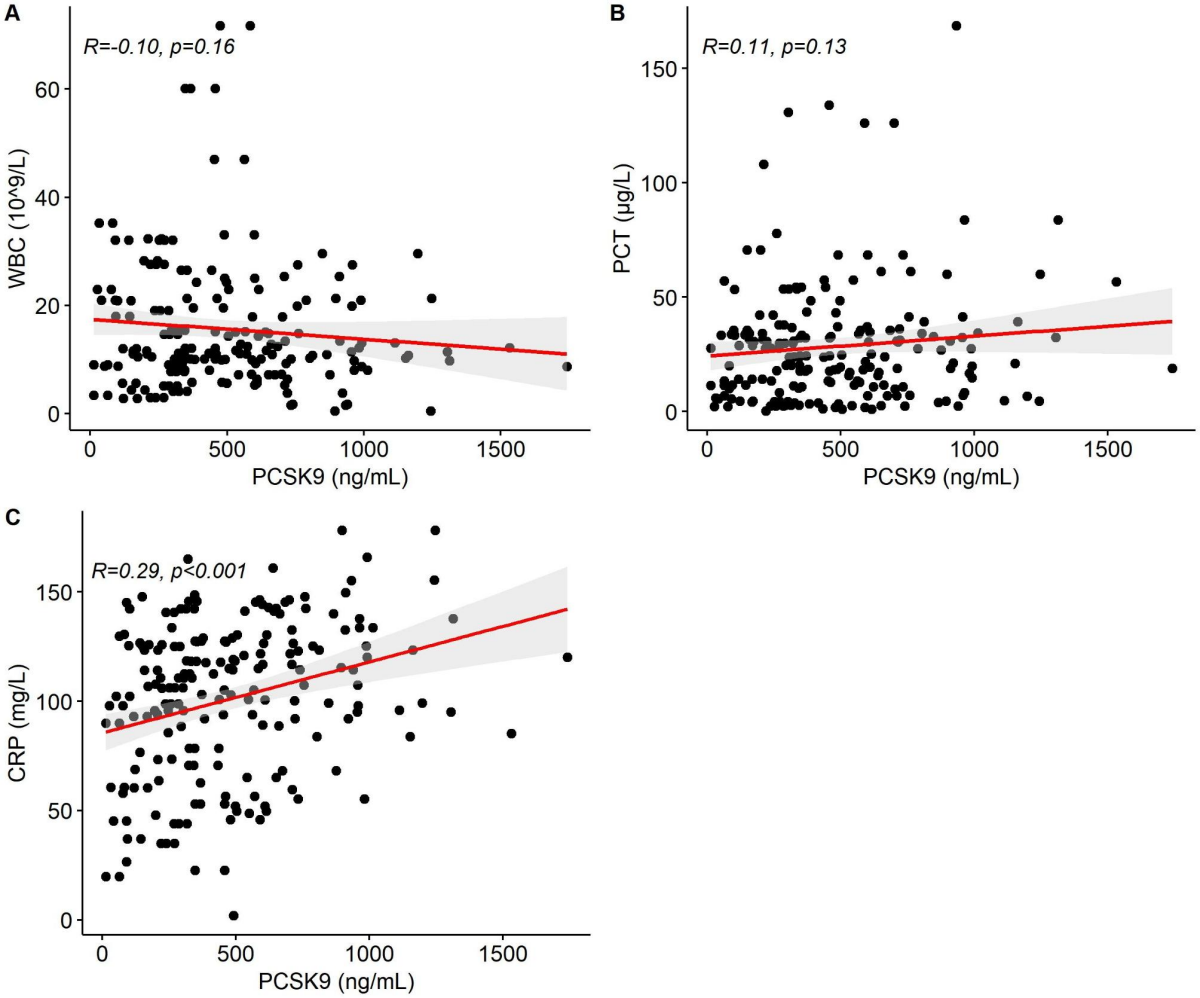
Associations between PCSK9 and WBC **(A)**, PCT **(B)**, and CRP **(C)** levels. The association was calculated using Pearson’s correlation coefficient (R). The grey area shows the 95% confidence interval (CI).

Pearson correlation analysis did not observe significant correlations of circulating PCSK9 level with white blood cells (WBC) (correlation coefficient R = -0.10, *p* = 0.16) and procalcitonin (PCT) (correlation coefficient R = 0.11, *p* = 0.13). However, the circulating PCSK9 level was positively correlated with C-reactive protein (CRP) (correlation coefficient R = 0.29, *p* < 0.001) (Fig. 2). Moreover, no significant association was found between PCSK9 and lipid indexes (Additional file 1: Fig. S1).

### Association between circulating PCSK9 and 28-day mortality of sepsis

**Fig. 3:**
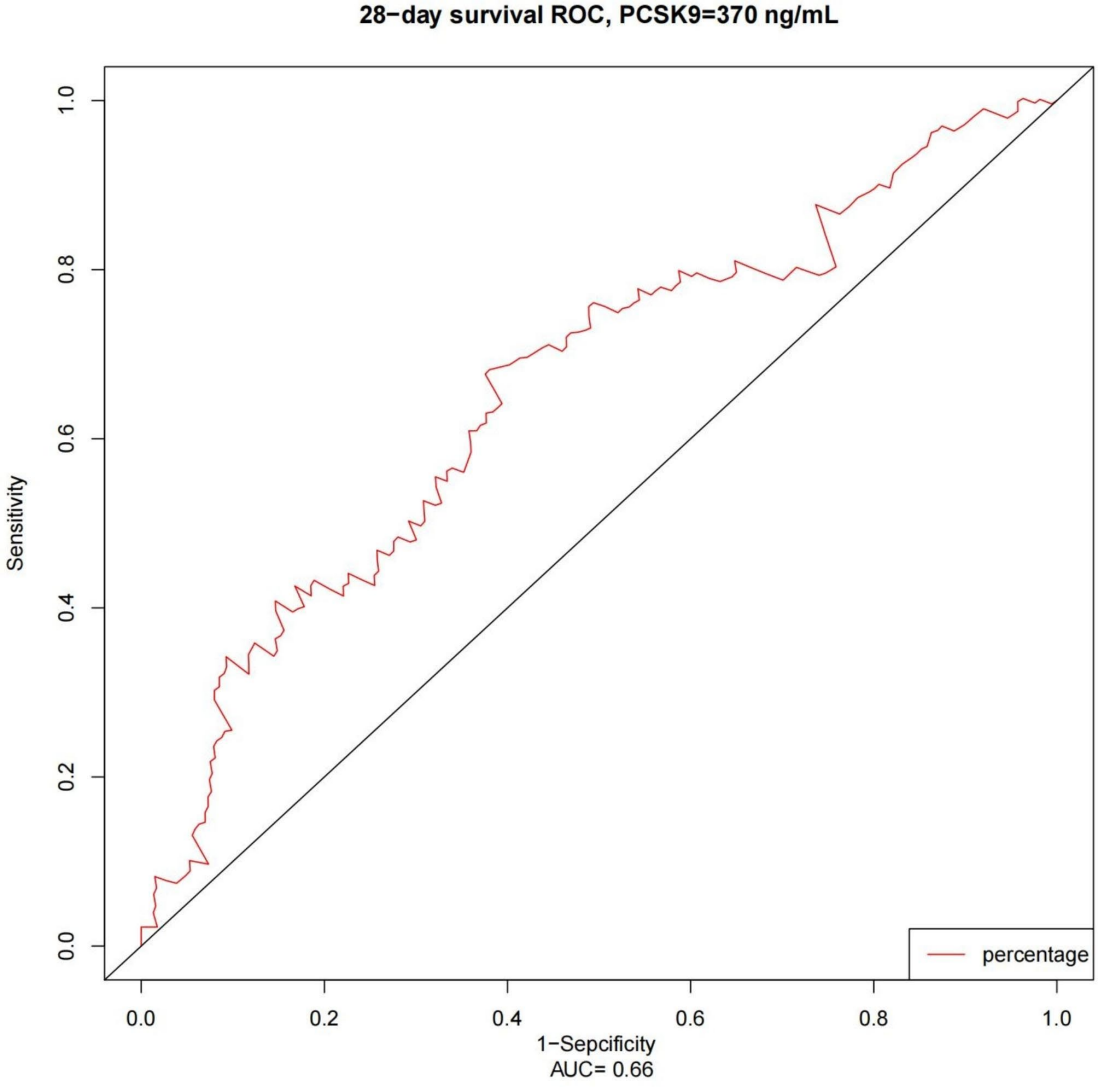
Time-dependent ROC curve of PCSK9 for overall survival at 28 days after the start of follow-up. The area under the ROC curve was 0.66. At a cut-off value of 370 ng/ml, PCSK9 achieved a sensitivity of 68.17% and a specificity of 61.97% in predicting sepsis survival, according to the Youden index

**Fig. 4:**
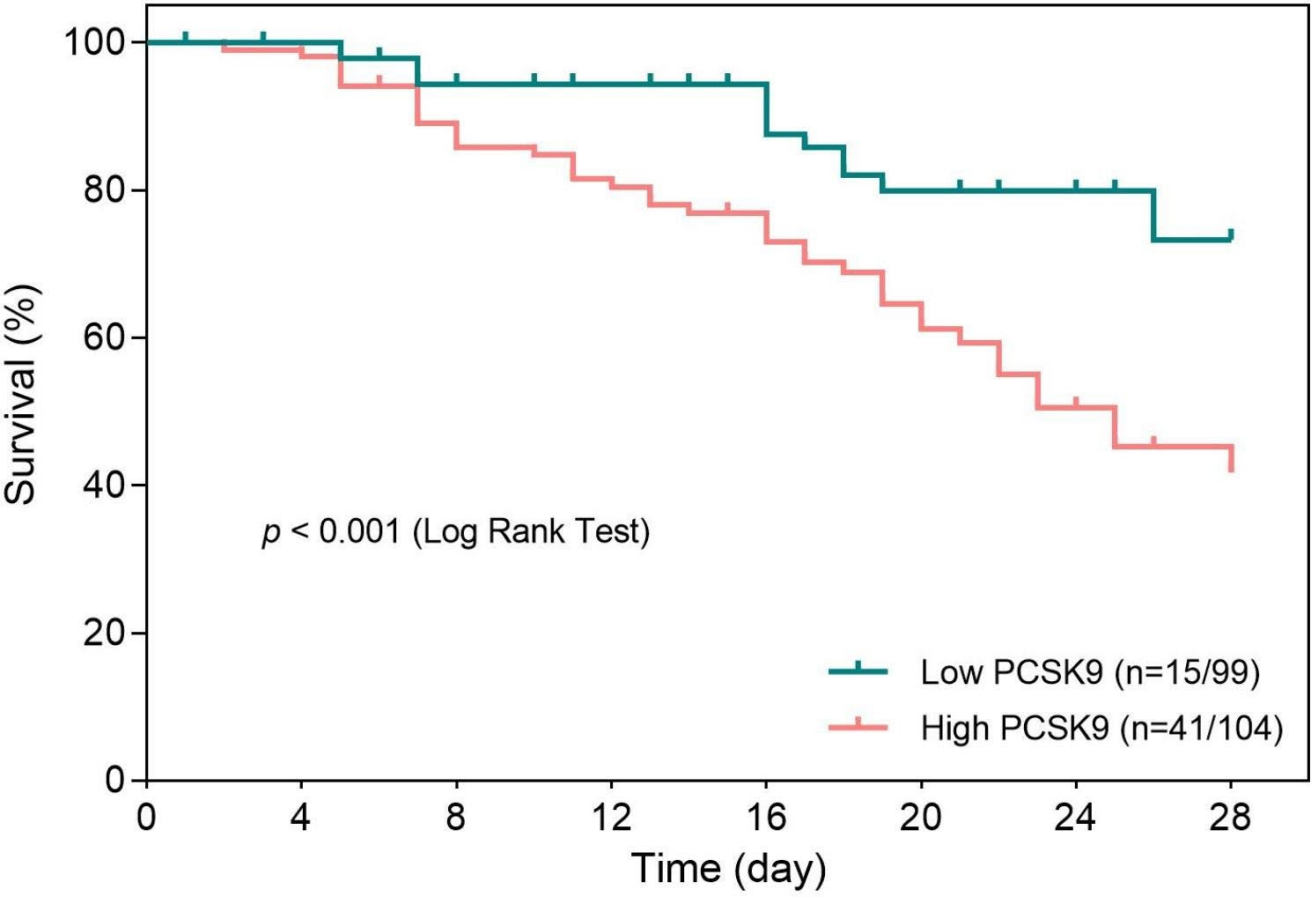
Cumulative overall survival curves stratified by PCSK9. Cumulative overall survival rates at 7, 14, 21, and 28 days were 94.35%, 94.35%, 79.94%, and 73.27% in patients with a low-PCSK9 group (≤ 370 ng/ml) and 89.03%, 76.85%, 59.34%, and 41.72% in a high-PCSK9 group (> 370 ng/ml) (*p* < 0.001, log-rank test)

Figure 3 shows the ROC curve for PCSK9 with overall survival at 28 days after the start of follow-up based on time-dependent ROC analysis. The optimal PCSK9 cut-off value was determined to be 370 ng/ml based on the Youden index. The Kaplan-Meier analysis of 28-day overall survival showed a significant difference between high- and low-PCSK9 groups (*p* < 0.001, Fig. 4).

The Cox proportional-hazards model revealed that the high-PCSK9 group represented a higher risk of 28-day mortality than the low-PCSK9 group, HR was 2.69 with 95% CI (1.49–4.87) in model 1 (unadjusted), 2.58 with 95% CI (1.37–4.84) in model 2 (after adjusted for age, sex, and comorbidities), and 2.56 with 95% CI (1.25–5.23) in model 3 (after adjusted for age, sex, comorbidities, SOFA, ALT, creatinine, BUN, hemoglobin, lactate, and TG) (Table 2).

**Table 2:** Associations between PCSK9 levels and 28-day all-cause mortality in patients with sepsis

Models	HR (95% CI)	*p*
Model 1 high-level vs. low-level	2.69 (1.49,4.87)	0.001
Model 2 high-level vs. low-level	2.58 (1.37,4.84)	0.003
Model 3 high-level vs. low-level	2.56 (1.25,5.23)	0.010

Model 1: unadjusted. Model 2: adjusted for age, sex, and comorbidities. Model 3: adjusted for age, sex, comorbidities, SOFA, ALT, creatinine, BUN, hemoglobin, lactate, and TG

## Discussion

This study, for the first time, found that circulating PCSK9 has a high prognostic value in Chinese sepsis patients. The cut-off value of serum PCSK9 level for predicting 28-day mortality was 370 ng/ml. According to this value, the high-PCSK9 group presented a much higher risk of 28-day mortality.

Causal relationships between PCSK9 level and the severity of sepsis are supported by clinical, experimental, and genetic studies. Pathogenic lipids (e.g. lipopolysaccharide, LPS) stimulate PCSK9 expression via SREBP2 pathways [16]. In a mouse model [17], PCSK9 overexpression aggravated LPS-induced sepsis, while knockout alleviated it [18]. Epidemiological data also confirm that levels of PCSK9 are elevated in patients with inflammatory diseases, particularly those with sepsis [19]. Moreover, in septic patients with coronavirus disease 2019 (COVID-19) with a more severe inflammatory response, PCSK9 levels are further elevated [20]. WBC, PCT, and CRP are commonly used diagnostic and prognostic biomarkers for sepsis [19, 21]. Consistent with our findings, it has been observed in a sepsis cohort that includes COVID-19 that there is a positive correlation between PCSK9 and CRP, but not with WBC and PCT [20]. This correlation was also observed in bacteremia patients [10]. However, this is at odds with the results of the study involving stable coronary heart disease patients who were naïve to lipid-lowering therapy [22], they reported a positive correlation between PCSK9 and WBC, which might be due to the difference existing in the study population.

Previous studies have revealed that serum triglyceride levels in septic patients were elevated but the cholesterol levels were decreased [23]. Importantly, decreased cholesterol levels may be associated with increased mortality in sepsis [24]. In the current study, it was found that triglyceride levels were higher in the survival group than in the death group, but the differences in cholesterol levels between the two groups were not significant, these results were consistent with previous studies [25]. Furthermore, though some studies reported positive correlations between circulating PCSK9 and lipid levels in healthy populations and cardiovascular patients [26, 27], these relationships were not significant in septic patients. Lipid metabolism was complex under an inflammatory state, inflammatory factors play an important role in the regulation of lipid metabolism, and serum lipid levels are shared largely variations in patients with inflammatory disease [28]. A study demonstrated that the serum levels of total cholesterol (TC), low-density lipoprotein cholesterol (LDL-C), and triglycerides (TG) were decreased in autoimmune rheumatic disease, and then increased after anti-inflammatory therapies [29].

A genetic study has demonstrated that patients carrying loss-of-function (LOF) alleles exhibit a higher rate of LPS clearance [30]. Experimental studies using genetic mouse models also showed similar results [31]. These results support that PCSK9 may play a central role in the pathogenesis or progression of sepsis. Theoretically, PCSK9 may predict the prognostic prediction of sepsis. Time-dependent ROC analysis showed the cut-off value of circulating PCSK9 level was 370 ng/ml. A previous study has found that the risk of multiple organ failure increases significantly in patients with PCSK9 levels > 370 ng/ml [9]. The multi-organ dysfunction is a major cause of death in septic patients. Therefore, the cut-off value set in the present study is sure of great clinical significance. Using the cut-off value to divide the patients into high- and low-PCSK9 groups, the high-PCSK9 group presented a much higher risk of 28-day mortality. After adjusting for potential confounders, this association remained evident. Although different studies of different populations have harvested inconsistent results, this study adds strong evidence to define the prognostic value of PCSK9 for mortality in sepsis patients.

This study has several limitations. First, some biases might remain in this single-center cohort study, although the data quality was controlled seriously. Second, the sample size was relatively small. However, the sample size might be sufficient according to a previous study calculation [12]. Moreover, gender, age, and cardiovascular disease may be confounding factors in observational studies, but these potential confounders exhibit no significant differences between the two groups. Finally, similar to previous studies, this study did not assess the association between longitudinal PCSK9 changes and prognosis, despite that PCSK9 changes during sepsis have been reported in several studies [9, 11].

## Conclusions

This study demonstrated that PCSK9 levels at or above 370 ng/ml significantly increase the risk of 28-day mortality in sepsis, providing evidence to evaluate the potential clinical significance of circulating PCSK9 in assessing the prognosis of septic.

### Electronic supplementary material

Below is the link to the electronic supplementary material.

Supplementary Material 1

Supplementary Material 2

## Data Availability

The data that support the findings of this study are available from the corresponding author (Chengfeng Qiu) but restrictions apply to the availability of these data, which were used under license for the current study, and so are not publicly available. Data are however available from the authors upon reasonable request and with permission of the corresponding author (Chengfeng Qiu).

## Abbreviations

ALT: alanine aminotransferase
AST: aspartate aminotransferase
BUN: blood urea nitrogen
CHD: coronary heart disease
CKD: chronic kidney disease
CRP: C-reactive protein
HDL-C: high-density lipoprotein cholesterol
HRs: hazard ratios
IQR: interquartile range
LDL-C: low-density lipoprotein cholesterol
LDLR: low-density lipoprotein receptor
LOF: loss-of-function
LPS: lipopolysaccharide
PCSK9: proprotein convertase subtilisin/kexin type-9
PCT: procalcitonin
RBC: red blood cell
ROC: receiver operating characteristic
SOFA: Sequential Organ Failure Assessment
TC: total cholesterol
TG: triglyceride
UA: uric acid
WBC: white blood cells
