# Supplementary material for: Elevated circulating PCSK9 level is associated with 28-day mortality in patients with sepsis: a prospective cohort study
Source: BMC Emerg Med. 2023 Oct 31;23:127. doi: 10.1186/s12873-023-00896-6 (PMC10617046; doi:10.1186/s12873-023-00896-6)
Supplement: Supplementary file 1 — Supplementary Material 1 [file 12873_2023_896_MOESM1_ESM.doc]

Additional file 1: Fig. S1. Associations between PCSK9 levels and lipid indexes. a, TC; b, HDL-C; c, LDL-C; d, TG.
