# Supplementary figures and images for: Elevated circulating PCSK9 level is associated with 28-day mortality in patients with sepsis: a prospective cohort study
Source: BMC Emerg Med. 2023 Oct 31;23:127. doi: 10.1186/s12873-023-00896-6 (PMC10617046; doi:10.1186/s12873-023-00896-6)

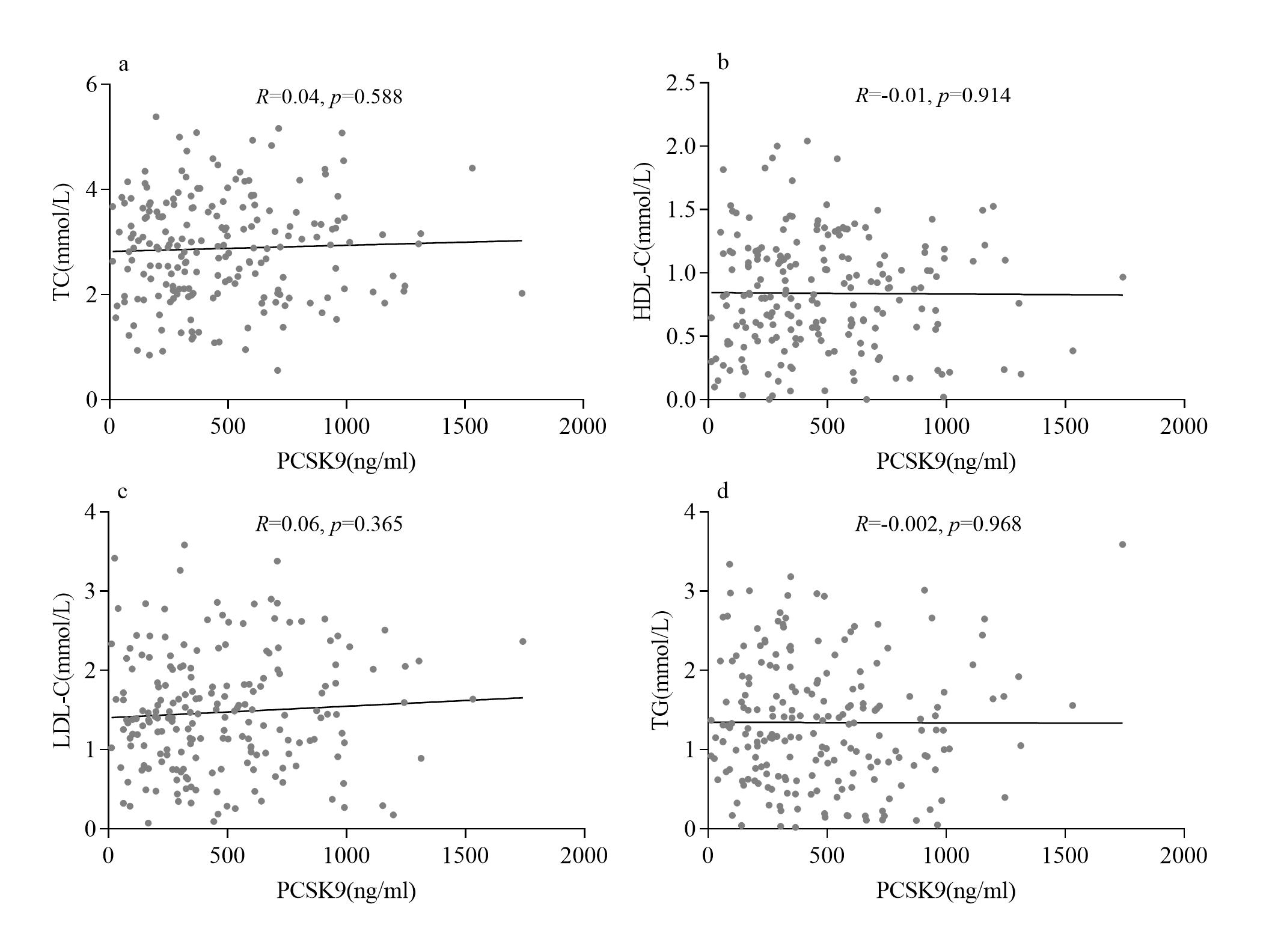

Supplement: Supplementary file 2 — Supplementary Material 2 [file 12873_2023_896_MOESM2_ESM.jpg]
